# Targeting colorectal cancer cell metabolism through development of cisplatin and metformin nano-cubosomes

**DOI:** 10.1186/s12885-018-4727-5

**Published:** 2018-08-15

**Authors:** Mona M. Saber, Abdulaziz M. Al-mahallawi, Noha N. Nassar, Björn Stork, Samia A. Shouman

**Affiliations:** 1 Department of Pharmacology and Toxicology, Faculty of Pharmacy, Cairo University, Kasr El-Aini St, Cairo, 11562 Egypt; 2 Institute of Molecular Medicine I, Medical Faculty, Heinrich-Heine-University, Universitätsstr. 1, Building 23.12, 40225 Düsseldorf, Germany; 3 Department of Pharmaceutics and Industrial Pharmacy, Faculty of Pharmacy, Cairo University, Kasr El-Aini St, Cairo, 11562 Egypt; 4 Pharmacology Unit, Department of Cancer Biology, National Cancer Institute, Cairo University, Kasr El-Aini St., Fom El Khalig, Cairo, 11796 Egypt

**Keywords:** AMPK, Cisplatin, Metformin, mTOR, Nano-cubosomes

## Abstract

**Background:**

Colorectal cancer (CRC) remains a leading cause of death worldwide. Utilizing cisplatin in CRC is correlated with severe adverse effects and drug-resistance. Combined anticancer drug-treatment, along with, their enhanced delivery, can effectively kill cancer through multiple pathways. Nano-cubosomes are emerging as nanocarriers for anticancer therapies, hence, we constructed nano-cubosomes bearing cisplatin and cisplatin-metformin combination for investigation on HCT-116 cells.

**Methods:**

Nano-cubosomes bearing either cisplatin alone or cisplatin-metformin combination were formulated using emulsification technique. The loaded nano-cubosomes were characterized in vitro and the optimized formulation was selected. Their cytotoxic effects were investigated by Sulphorhodamine-B (SRB) assay. The AMPK/mTOR metabolic pathway as well as the Akt/mTOR pathway were analyzed using ELISA technique. Colorimetry was used in NADPH oxidase, LDH and caspase-3 activity determination.

**Results:**

nano-cubosomal formulations exhibited superior cytotoxic effect compared to unformulated cisplatin. This cytotoxic effect was profound upon incorporation of metformin, an indirect mTOR inhibitor, in cisplatin nano-cubosomes. The induced CRC cell apoptosis was through inhibition of several metabolic pathways, namely, AMPK/mTOR and Akt/mTOR. Drug-loaded nano-cubosomes ensued depletion in glucose and energy levels that led to AMPK activation and thus mTOR inhibition. mTOR was additionally inhibited via suppression of p-Akt (Ser473) levels after nano-cubosomal treatment. Moreover, drug-loaded nano-cubosomes produced a notable escalation in ROS levels, evident as an increase in NADPH oxidase, inhibition of LDH and a consequential upsurge in caspase-3.

**Conclusion:**

These results demonstrated the influence exerted by cisplatin-loaded nano-cubosomes on CRC cell survival and enhancement of their cytotoxicity upon metformin addition.

## Background

Colorectal cancer (CRC) is one of the most common causes of cancer-related death worldwide, with over a million newly diagnosed cases per year. Although, cisplatin is designated as a golden chemotherapeutic agent in solid tumor treatment, in CRC, therapy is accompanied by dose-limiting adverse effects, resistance and decreased effectiveness. Literature suggests that cisplatin effectiveness as a chemotherapeutic agent in CRC could be improved by combinatorial approaches [1].

Metformin received increased attention for its potential anti-tumorigenic effects which are independent of its blood glucose lowering action; notably, studies revealed lower CRC incidence and mortality with its use [2]. On CRC cell lines, it inhibited cell growth and synergistically increased apoptosis when combined with other chemotherapy drugs [3]. One proposed antitumor mechanism of metformin is reprogramming of cancer cell metabolism that results in forceful effects on gene expression, cell differentiation, proliferation and tumor microenvironment [4].

Advances in chemotherapy treatment of CRC are limited to the currently available selection of licensed drugs, most of which have been used for many years. The slow progress in disease treatment resulted in an increased need for a new approach like nano-systems to overcome problems of drug delivery. Nano-systems use in cancer offer the possibility to enhance drug efficacy with minimal side effects. Despite the remarkable development of different nanoparticle types for various purposes, relatively little is known about the cellular interactions and toxicity of monoolein-based nano-cubosomes. Several nanoparticle preparations containing cisplatin were formulated, including liposomes, gold nanoparticles and dendrimers, however, the anticipated improvement in efficacy was not observed [5]. To this end, the current study aimed to formulate nano-cubosomes (self-assembled cubic liquid crystalline nanoparticles) incorporating cisplatin and metformin in an attempt to increase efficacy in HCT-116 CRC cells, as well as, investigating the possible effect on tumorigenesis-associated metabolism.

## Methods

### Drugs

Cisplatin and metformin were obtained from Mylan (Virginia, USA) and CID Co. (Cairo, Egypt). The drugs were freshly dissolved in saline and all experiments carried out protected from light.

### Chemicals

Roswell Park Memorial Institute-1640 (RPMI-1640), glyceryl monooleate (GMO), polyvinyl alcohol (PVA), Pluronic-F127 and sulphorhodamine-B (SRB) were obtained from Sigma Chemical Co. (St. Louis, USA). All other chemicals and reagents used were of analytical grade and used without further purification.

### Preparation of nano-cubosomal dispersions

**Fig. 1:**
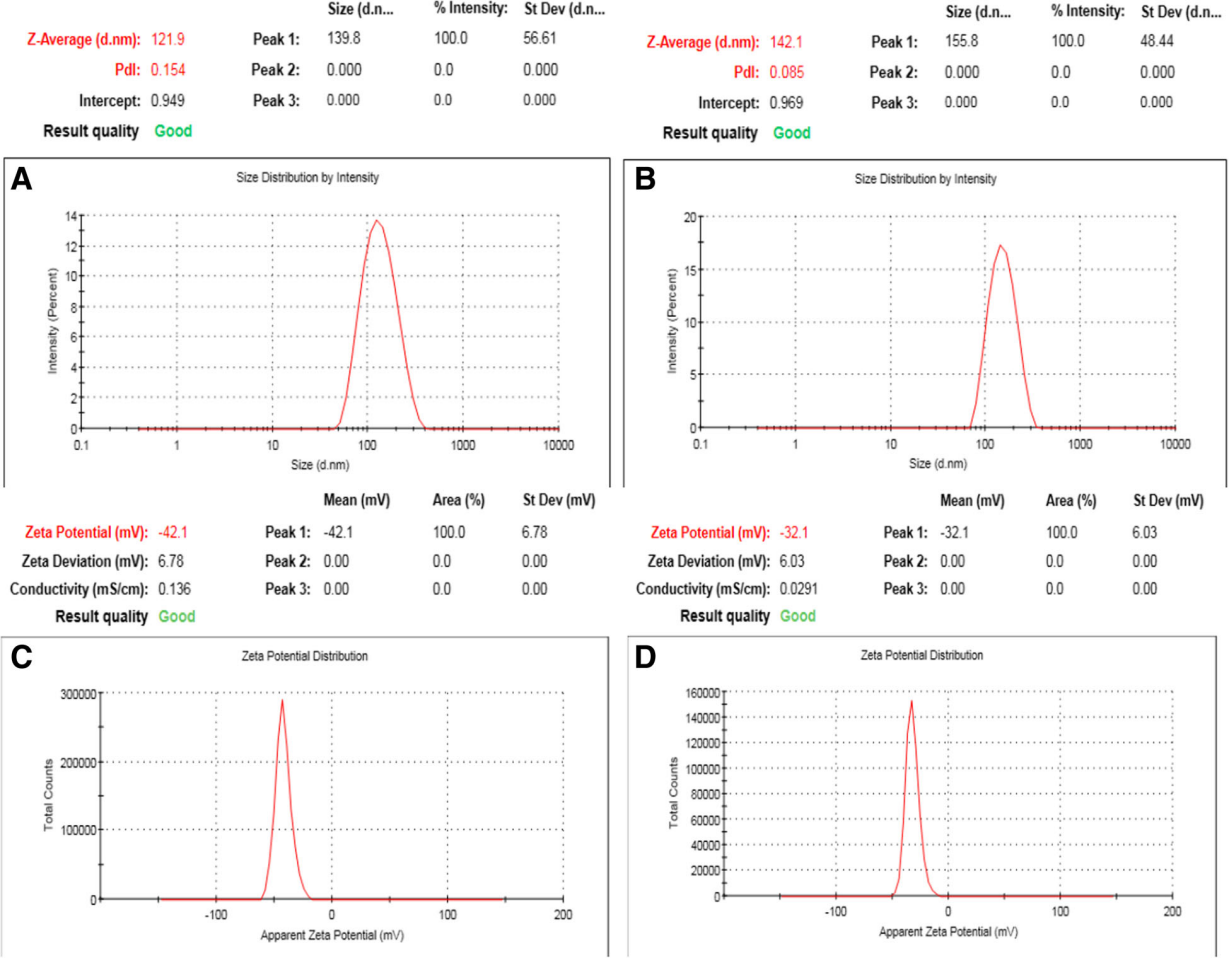
Particle size distribution and zeta potential of **a**, **c** cisplatin nano-cubosomes and **b**, **d** cisplatin-metformin nano-cubosomes

**Fig. 2:**
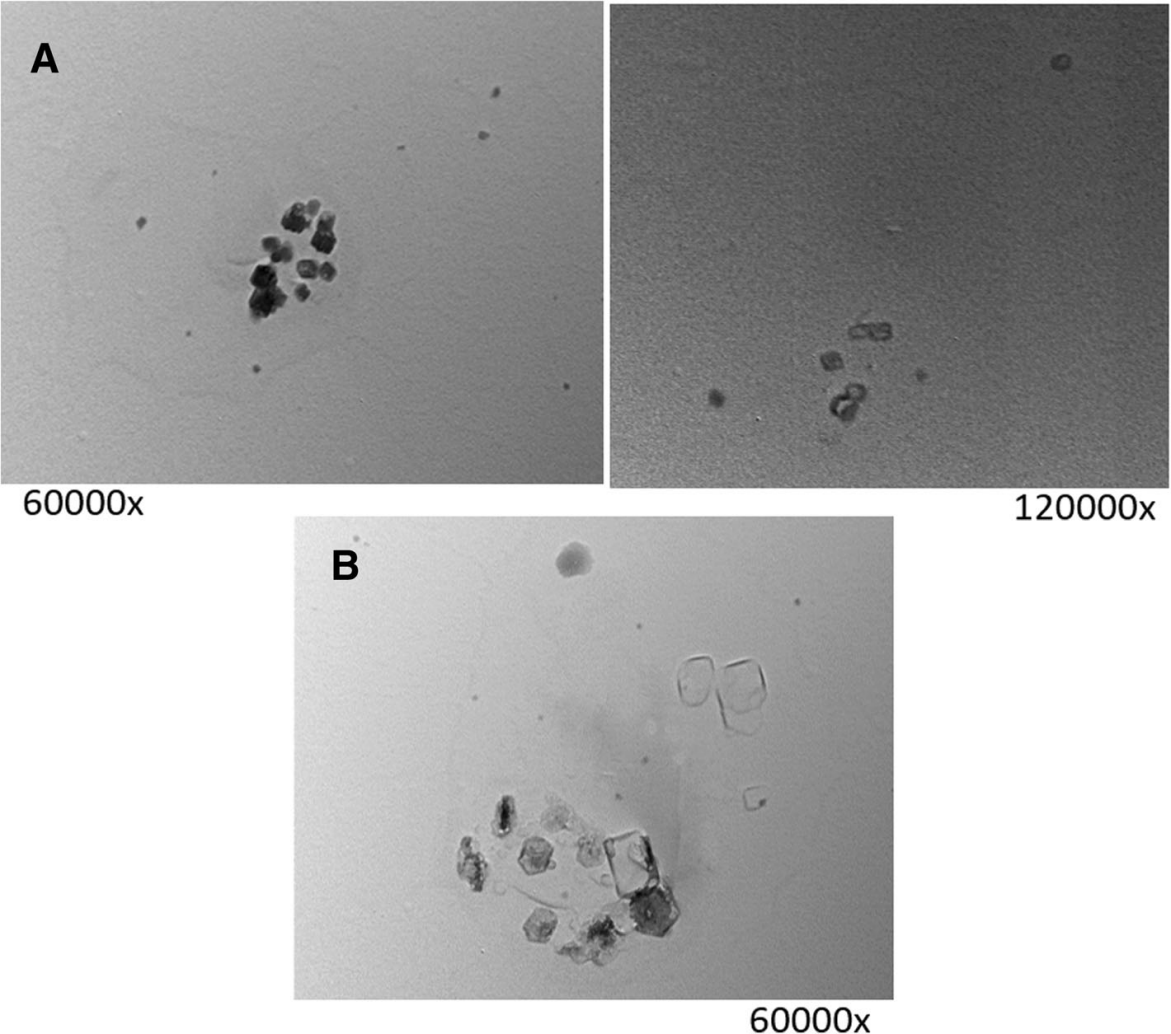
Transmission electron micrographs of different nano-cubosomal dispersions; **a** cisplatin nano-cubosomes and **b** cisplatin-metformin nano-cubosomes

Nano-cubosomal dispersions with average particle size ranging from 120 nm to 150 nm and polydispersity index from 0.05 to 0.20 were prepared. The prepared nano-formulations exhibited zeta potential from − 30 mV to − 45 mV indicating that they have sufficient charges that would inhibit their aggregation [6] (Fig.1). The nano-cubosomes were prepared by emulsification technique described by Morsi et al. [7] with slight modifications. For blank nano-cubosomes, GMO and Pluronic-F127 were melted at 70 °C. The obtained molten solution was added drop-wise to 20 mL normal saline (70 °C) containing 2.5% PVA under mechanical stirring at 1500 rpm. Dispersions were maintained under stirring and cooled to room temperature to achieve homogenous state followed by probe sonication for 10 min (at 70% amplitude) [8]. For the preparation of drug-loaded nano-cubosomes, the drugs (cisplatin or cisplatin-metformin combination) were added to the aqueous phase prior to addition of the molten hydrophobic phase. The final cisplatin concentration in the nano-cubosomal dispersion was 60 μM. Formulas were freshly prepared before each experiment. Transmission electron microscope (TEM) revealed that the prepared cubosomes are in the nano-size, which confirms the results of particle size measurement, and showed nanoparticles of cubical nanostructure (Fig.2).

### Cell culture

Human CRC cell line, HCT-116 (ATCC® CCL-247™), was obtained from the American Type Culture Collection (Manassas, USA). At the Egyptian National Cancer Institute (Cairo, Egypt), it was maintained and grown in RPMI-1640 supplemented with 10% fetal bovine serum, 1.5 g/L sodium bicarbonate, 2 mM L-glutamine and 1% penicillin/streptomycin in 5% CO_2_ at 37 °C.

### Cytotoxicity assay

SRB assay was used to evaluate cytotoxicity. Exponentially growing cells were seeded in 96-well plates at an initial density of 5 × 10^3^/well. Nano-cubosomes were added after 24 h with various concentrations and incubated at 37 °C for 48 h to determine their IC_50_s (the concentration of the drug required to produce 50% cell growth inhibition). Cells were fixed with trichloroacetic acid (10%) for 1 h at 4 °C, stained with SRB (0.4%) for 30 min., then washed four times with acetic acid (1%) and air-dried. The dye was dissolved with 10 mM Tris base (pH 10.5) before measuring the optical density (O.D.) spectrophotometrically at 570 nm with the microplate reader (Tecan SunriseTM, Switzerland). Cell survival fraction was calculated as follows: survival fraction = O.D. (treated cells)/O.D. (control cells). The IC_50_s after 48 h treatment were calculated using sigmoidal dose-response curve-fitting models (Graphpad Prism Software, version 5.03, USA). The concentration of cisplatin nano-cubosomes that inhibited 30% of the cells was determined and used to prepare different nano-cubosomal formulations containing fixed IC_30_ of cisplatin and different metformin concentrations. This was done to identify the metformin concentration that will produce an IC_50_ when used with cisplatin. The concentrations used in all experiments were the IC_30_ of the cisplatin nano-cubosomes (7 μM), cisplatin (7 μM)-metformin (7 mM) nano-cubosomes and the same concentration of the single drugs as their respective controls.

### Evaluation of drug interaction

Concentration response curves of metformin and cisplatin alone in HCT-116 cells were first generated. The interaction between metformin and cisplatin in nano-cubosomes was then analyzed by calculating the combination index (CI) using the following isobologram equation:

$$ \mathrm{CI}=\mathrm{d}1/\mathrm{D}1+\mathrm{d}2/\mathrm{D}2 $$

Where d1 and d2 are the respective concentrations of the drugs used in the combination required to produce a fixed level of inhibition while D1 and D2 are their concentrations able to produce alone the same magnitude of effect. If CI is less than 1, the interaction between the two drugs is synergistic, while if CI = 1 or > 1, the interaction is additive or antagonistic, respectively.

### Determination of cisplatin levels

After treating HCT-116 cells with cisplatin, cisplatin nano-cubosomes and cisplatin-metformin nano-cubosomes for 48 h, cells were collected, washed with PBS solution, lysed, centrifugated and supernatant separated to obtain a clear cell lysate. Cisplatin levels were determined according to the method developed by Golla and Ayres which is based on complexing cisplatin with o-phenylenediamine to give a green color [9]. The product was obtained at pH 6.2, in 30 min at 90 °C, giving a maximum absorbance at 705 nm.

### Measurement of glucose, ATP and lactate levels

After treatment of cells with desired concentrations of metformin, cisplatin, cisplatin nano-cubosomes and cisplatin-metformin nano-cubosomes for 48 h, the medium was collected for glucose level analysis and cells were harvested and washed in PBS for ATP level measurement. Glucose level was detected using Spinreact glucose-TR kit (Santa Coloma, Spain) based on its oxidation to gluconic acid by glucose oxidase. The formed hydrogen peroxide, is detected by a chromogenic oxygen acceptor. The intensity of the color formed is measured at 505 nm and is proportional to glucose concentration which was calculated with reference to a glucose standard solution.

ATP levels were measured in the cell lysate using ATP colorimetric assay kit (BioVision, USA) which utilizes the phosphorylation of glycerol to generate a product that is quantified colorimetrically at 570 nm.

Lactate levels in the cell culture medium were measured using L-lactate colorimetric assay kit (Abcam, U.K) where lactate is oxidized by lactate dehydrogenase to generate a product that interacts with a probe to produce a color that is measured at 450 nm.

### Assessment of AMP/ATP ratio

To further investigate the energy status of the HCT-116 cells, AMP/ATP ratios after 24 h and 48 h were measured. The cells were treated with metformin, cisplatin, cisplatin nano-cubosomes and cisplatin-metformin nano-cubosomes, then harvested and washed with PBS. The AMP/ATP ratios were determined using ATP/ADP/AMP assay kit (Biomedical Research Service, NY, USA) according to the manufacturers’ instructions.

### Intracellular lactate dehydrogenase (LDH) activity measurement

Cells were treated with metformin, cisplatin and drug-loaded nano-cubosomal preparations for 48 h then collected and washed with PBS. Lactate dehydrogenase enzyme catalyzes the conversion of pyruvate, the end product of glycolysis, to lactate with the recycling of NADH back to NAD^+^. In this assay, the rate of decrease in NADH concentration is measured and is proportional to LDH concentration. NADH interacts with a specific probe to produce a color measured at 340 nm.

### NADPH oxidase activity measurement

Cells were grown in 75 cm^2^ flasks and allowed to adhere for 24 h then treated with the different treatment groups for 48 h. Cells were then collected by trypsinisation and the cell pellet was washed twice with PBS. NADPH oxidase activity was measured using cytochrome-c reductase NADPH assay kit (Sigma, USA). The method depends on measurement of cytochrome-c reduction by NADPH-cytochrome-c reductase in the presence of NADPH. The oxidation/reduction state of cytochrome-c alters the absorption spectrum. Cytochrome-c reduction is monitored by the increase in its absorbance at 550 nm.

### Total AMPK, p002DAMPK, total mTOR, p-mTOR, total Akt and p-Akt protein level assessment

According to the kit manufacturer’s instructions, total AMPK, p-AMPK alpha (S487), total mTOR, p-mTOR (S2448), total Akt and p-Akt (S473) protein levels were determined using RayBiotech Kits (Georgia, USA). Cell lysate samples were pipetted into the wells of a microplate pre-coated with polyclonal antibody against human AMPK, mTOR or Akt. The measured biomarkers present in the solutions were bound by the immobilized antibody and a color, measured at 450 nm, is developed which is proportional to the amount of protein bound.

### Determination of Caspase-3 activity

Samples of equal protein concentrations were assayed colorimetrically (Caspase-3/CPP32, BioVision, USA) according to the manufacturer’s instructions to measure caspase-3 activity. The assay depends on cleavage of the peptide from the color reporter molecule p-nitroaniline (pNA) by caspase-3 and chromophore detection at 405 nm.

### Determination of protein content

Protein amounts were measured by the Bradford method using Coomassie Protein Assay Kit (Pierce, USA) and all the results were expressed per mg protein content [10].

### Statistical analysis

All values are expressed as mean ± SD from a minimum of three different experiments. One way analysis of variance (ANOVA) was used followed by Tukey-Kramer multiple comparison test to determine the level of statistical significance which was considered at *p* < 0.05. Statistical analysis was performed using Graphpad InStat, version 5.0 (Graphpad, USA).

## Results

### Enhanced cisplatin cytotoxicity after nano-cubosomal incorporation with metformin

**Fig. 3:**
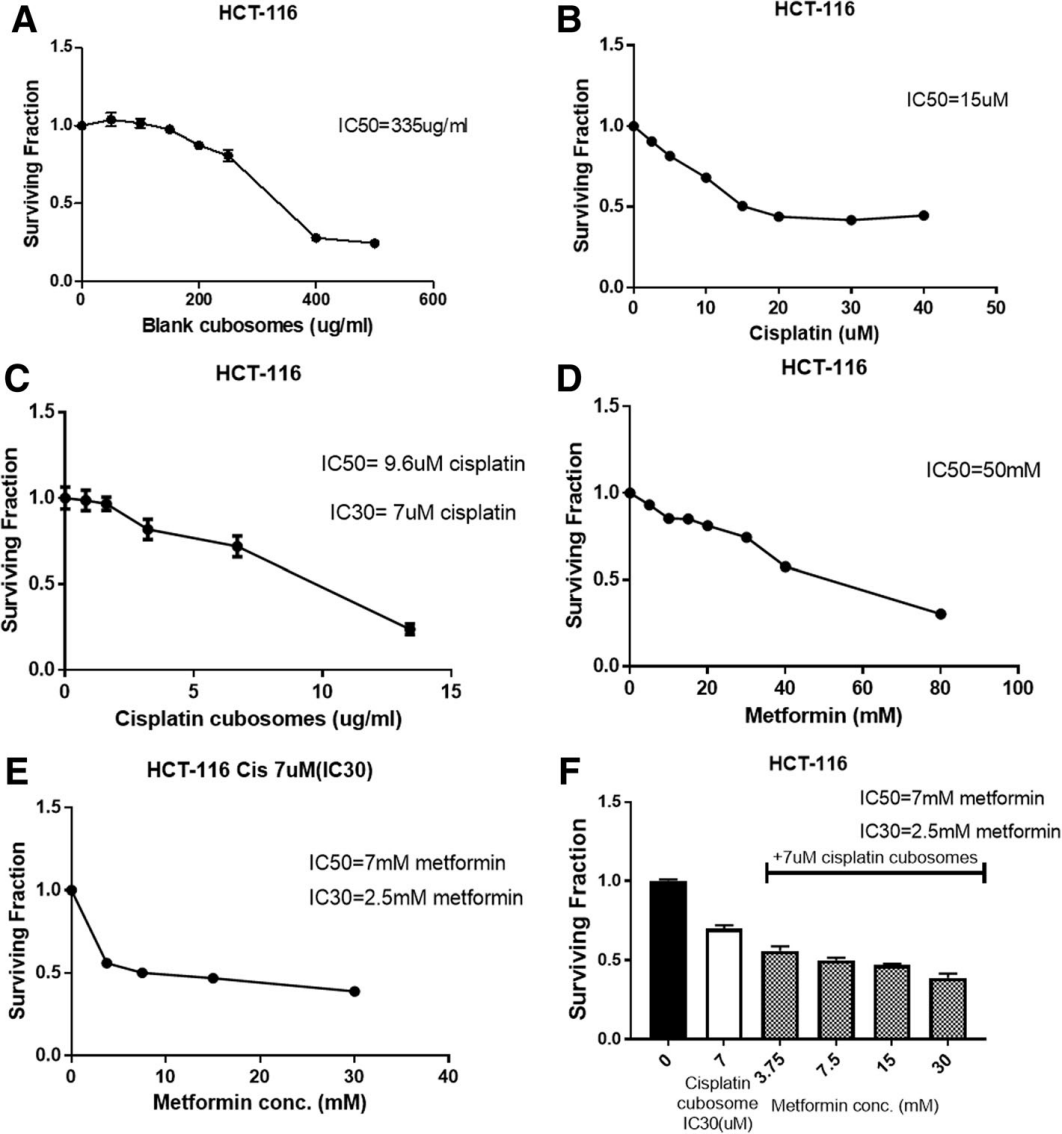
Surviving fraction and the IC_50_ of HCT-116 cells after 48 h treatment with **a** blank nano-cubosomes **b** cisplatin **c** cisplatin nano-cubosomes **d** metformin **e** cisplatin-metformin nano-cubosomes **f** IC_30_ cisplatin nano-cubosomes and different metformin concentrations. The actual data represent the mean ± SD of 3 separate experiments performed in sextuplets

Treatment of HCT-116 with cisplatin resulted in an IC_50_ of 15 μM, while cisplatin-loaded nano-cubosomes led to a decrease in IC_50_ to 9.6 μM. Moreover, incorporation of 7 mM metformin with IC_30_ cisplatin (7 μM) in nano-cubosomes inhibited cell growth by 50%. From the fraction of cell survival values, it is confirmed that the cytotoxic effects of the drug-loaded nanoparticles was higher than the effects of the individual drug (Fig. 3).

### Synergistic interaction between metformin and cisplatin in nano-cubosomes

**Fig. 4:**
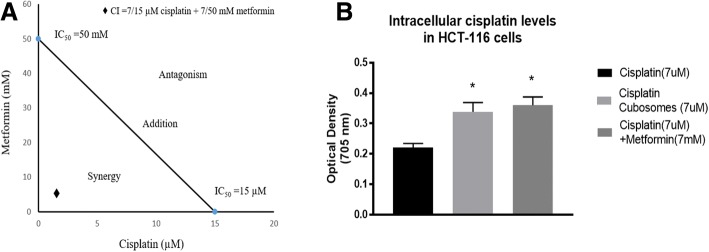
**a** Combination index of metformin and cisplatin. **b** Optical density of cisplatin in HCT-116 cells treated with cisplatin (7 μM), cisplatin nano-cubosomes (7 μM) or cisplatin (7 μM)-metformin (7 mM) nano-cubosome combination for 48 h. All data are expressed as mean ± SD of 3 separate experiments. The statistical significance of the results was analyzed using one way ANOVA followed by Tukey-Kramer multiple comparison test. ^*^Significantly different from cisplatin (7 μM) (*P* < 0.05)

According to the isobologram equation used for determination of the type of drug interaction, cisplatin and metformin were found to have a synergistic effect with a combination index of 0.606, less than 1, as shown in (Fig. 4a).

### Increased cisplatin intracellular accumulation after incorporation into nano-cubosomes

Measurement of intracellular cisplatin levels after 48 h of treatment with 7 μM cisplatin, 7 μM cisplatin nano-cubosomes and cisplatin (7 μM)-metformin (7 mM) nano-cubosomes indicated that incorporation into nano-cubosomes increased drug uptake. This was evident in the nano-cubosomes treated cells where a 1.5–1.6 fold increase in cisplatin levels was observed compared to unformulated cisplatin (Fig. 4b).

### Depletion of glucose and ATP following nano-cubosomal treatment

After 24 h of treatment a decrease in extracellular glucose level was detected in all treatment groups. Cisplatin nano-cubosomes and cisplatin-metformin nano-cubosomes showed the highest drop in glucose levels that reached 17 and 11 mg/dl compared to 42.5 mg/dl measured in the control group. An anticipated increase in the corresponding ATP levels was not observed. On the contrary all groups showed significant decrease in ATP which was prominent in the nano-cubosome containing groups.

**Fig. 5:**
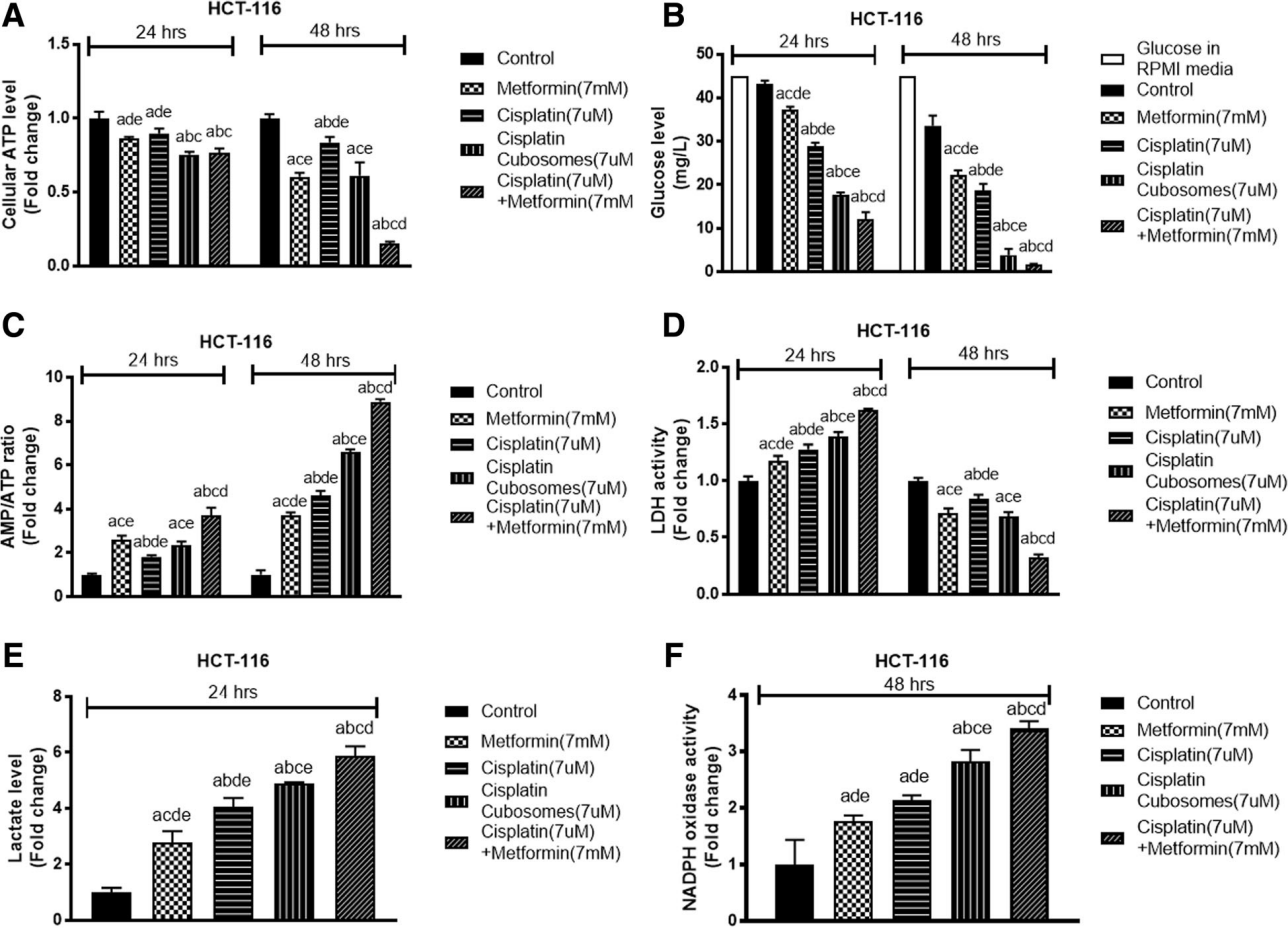
Levels of **a** cellular ATP, **b** glucose, **c** AMP/ATP ratio, **d** LDH activity, **e** lactate and **f** NADPH oxidase activity of HCT-116 cells. The cells were treated with metformin (7 mM), cisplatin (7 μM), cisplatin nano-cubosomes (7 μM) or cisplatin (7 μM)-metformin (7 mM) nano-cubosome combination for 24 h and/or 48 h. All data are expressed as mean ± SD of 3 separate experiments. The statistical significance of the results was analyzed using one way ANOVA followed by Tukey-Kramer multiple comparison test. ^a^Significantly different from control, ^b^from metformin ^c^from cisplatin ^d^from cisplatin nano-cubosomes ^e^ from cisplatin-metformin nano-cubosomes (*P* < 0.05)

Treatment of HCT-116 cells for 48 h with cisplatin resulted in ATP reduction in cell lysate by 18% accompanied by an increase in glucose uptake in an attempt to restore energy balance, leading to the decrease in its level in the medium by 60%. On the other hand, cisplatin nano-cubosomes decreased ATP by 40% complemented with a greater diminution of glucose from the cell culture medium that reached 90%. Addition of metformin to cisplatin nano-cubosomes resulted in an enhancement of the ATP reduction by 88% and a resultant glucose depletion reaching nearly undetectable levels (Fig. 5a and b).

### Increased AMP/ATP ratio in treated cells

Figure 5c shows an increase in AMP/ATP ratio marking an increase in ATP consumption or decreased synthesis. Metformin, cisplatin, cisplatin nano-cubosomes and cisplatin-metformin nano-cubosomes showed an increase of 2.5, 1.7, 2.15 and 3.6 fold after 24 h of treatment compared to the control.

A marked elevation in the AMP/ATP ratio was detected after 48 h of treatment. It reached 3.6, 4.5, 6.5 and 8.8 fold in metformin, cisplatin, cisplatin nano-cubosomes and cisplatin-metformin nano-cubosomes groups.

### Initial activation followed by inhibition of intracellular LDH with a consequent rise in extracellular lactate upon nano-cubosomal treatment

Figure 5d shows that treatment of HCT-116 cells with metformin or cisplatin for 24 h resulted in an increase in LDH activity of 1.15 and 1.25 folds respectively. Incorporation of cisplatin in nano-cubosomes increased LDH activity to 1.35 fold. Upon combination of both drugs, a 1.6 fold increase in the enzyme activity was observed that was statistically from all treatment groups. Coupled with the rise in enzyme activity an increase in extracellular lactate levels was detected with a similar pattern as shown in Fig. 5e.

After 48 h a shift in the enzyme activity was observed with a notable inhibition in all groups. Unformulated cisplatin decreased LDH activity by 17% while cisplatin nano-cubosomes decreased its activity by 33%. Upon addition of metformin to cisplatin nano-cubosomes, LDH inhibition reached 70%.

### Increased oxidative stress by cisplatin-metformin nano-cubosomes

As a measure of oxidative stress, NADPH oxidase activity was assessed in all treatment groups. The drug alone produced a 2 fold increase in NADPH oxidase activity compared to a 2.8 fold increase produced by cisplatin nano-cubosomes. The combined formula produced a 3.4 fold rise in the enzyme activity that was statistically significant from the cisplatin group only (Fig. 5f).

### Dual inhibition of mTOR activity via pronounced AMPK activation and p-Akt suppression

**Fig. 6:**
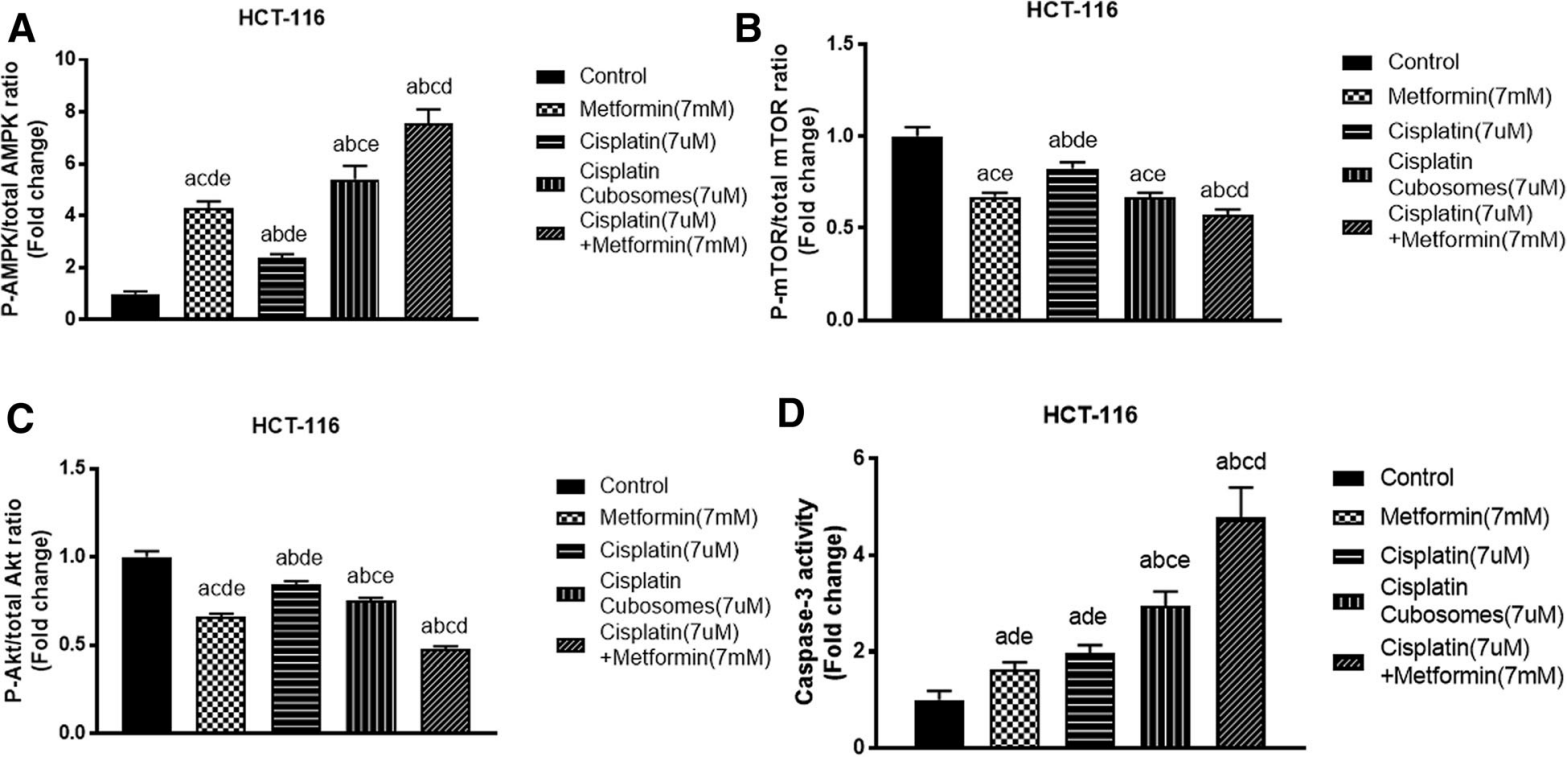
Fold change in intracellular **a** p-AMPK/AMPK ratio, **b** p-mTOR/mTOR ratio, **c** p-Akt/Akt ratio levels of HCT-116 cells treated for 24 h and **d** caspase-3 activity of HCT-116 cells treated for 48 h. The cells were treated with metformin (7 mM), cisplatin (7 μM), cisplatin nano-cubosomes (7 μM) or cisplatin (7 μM)-metformin (7 mM) nano-cubosome combination for 24 h. All data are expressed as mean ± SD of 3 separate experiments. The statistical significance of the results was analyzed using one way ANOVA followed by Tukey-Kramer multiple comparison test. ^a^Significantly different from control, ^b^from metformin ^c^from cisplatin ^d^from cisplatin nano-cubosomes ^e^ from cisplatin-metformin nano-cubosomes (*P* < 0.05)

The nano-cubosomes containing both drugs produced a 7.5 fold increase in p-AMPK accompanied by a reduction in p-mTOR levels by 45%. This was significantly different from the individual treatments, where metformin and cisplatin groups showed 4 and 2.25 fold increase compared to the control group. Cisplatin nano-cubosomes showed a 5.25 fold increase that was statistically significant from cisplatin only treatment. A consequent decrease in p-mTOR levels was noticed in all treatment groups with a similar pattern. Cisplatin treatment resulted in a 20% decrease, while metformin and cisplatin nano-cubosomes treatment groups produced a 35% decrease, respectively (Fig. 6).

To further determine if mTOR was inhibited by other pathways we analyzed the levels of p-Akt. Metformin addition to the cisplatin nano-cubosomes reduced phosphorylated Akt levels by 55% compared to the control. Cisplatin nano-cubosomes showed a robust inhibition of 28%, while cisplatin alone had a lower effect evident as 18% decrease.

### Enhanced Caspase-3 activity

As shown in Fig. 6d, administration of cisplatin and formulated cisplatin increased caspase-3 activity two and three folds over the normal control. Incorporation of metformin into cisplatin nano-cubosomes produced an exaggerated 4.8 folds increase in caspase-3 activity and apoptosis that was significant from all treatment groups.

## Discussion

Drug-loaded lipid-based systems have become an outstanding theme of research in therapeutics [11, 12]. Amongst these, the monoolein-based nano-cubosomes emerged as one of the most cost-effective and clinically promising technology in disease diagnosis and treatment [13, 14]. They possess a unique nanostructure consisting of a curved bilayer whose three-dimensional folding originates two disconnected, continuous water channels. This structure generates lipophilic and hydrophilic domains to integrate water-soluble, oil-soluble, and amphiphilic substances [15]. Apart from being biocompatible, biodegradable and lack of toxicity; it can incorporate drug amounts more than liposomes [16], as well as, protecting the drugs against physiological or chemical degradation [17].

Cisplatin is one of the platinum compounds used frequently in solid tumors. Due to the high rates of resistance in CRC, there were previous attempts to use cisplatin in the form of nanoparticles, showing that its efficacy and delivery to the tumor can be enhanced. However, little information is available on the cellular interaction of lipid-based cisplatin nanoparticles in vitro. In the present study, we show that cisplatin administration as nano-cubosomes alone or in combination with metformin demonstrated an exaggerated increase in CRC cell death using low drug concentrations.

**Fig. 7:**
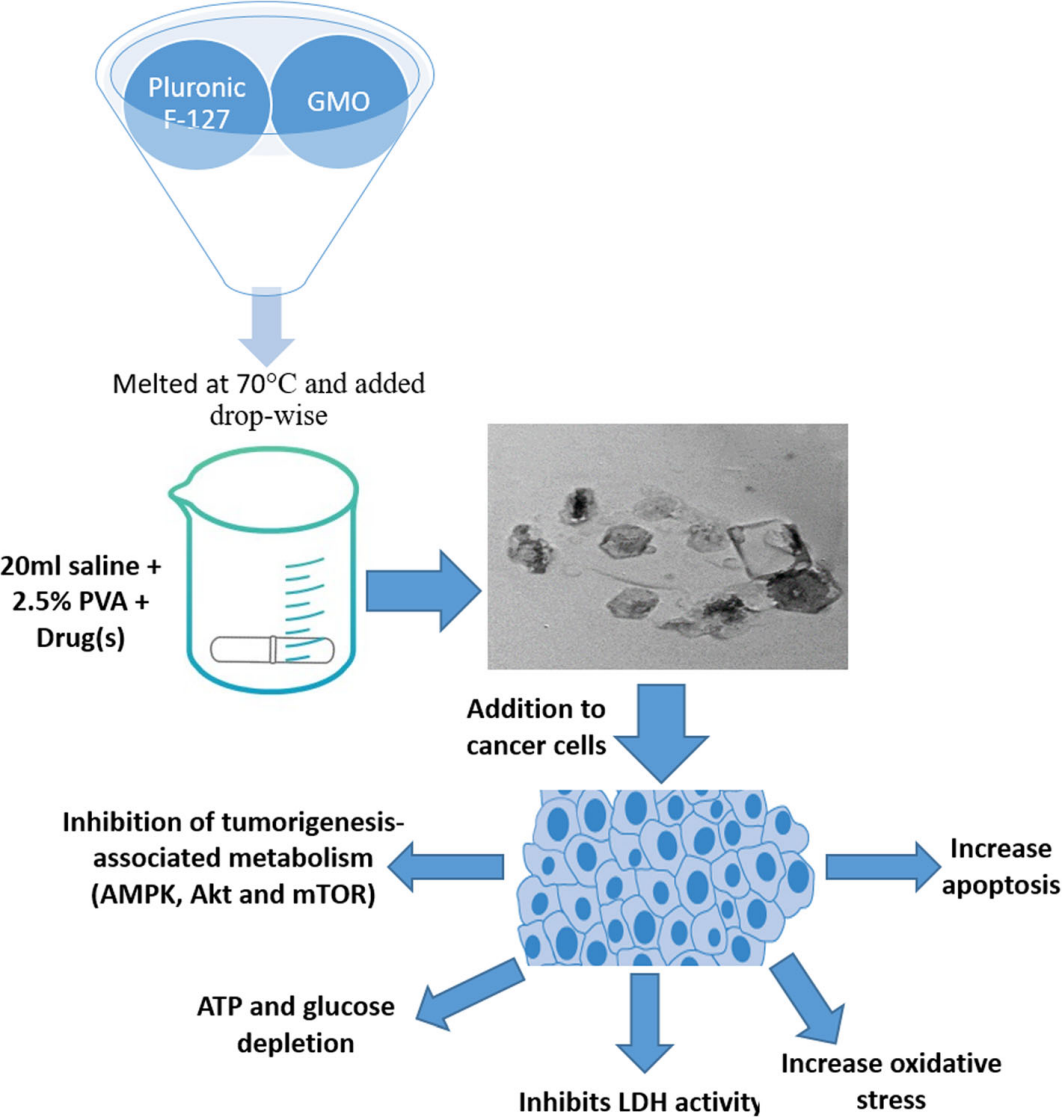
Preparation of cisplatin and cisplatin-metformin nano-cubosomes using the emulsification technique. Treatment of CRC cells with drug-loaded nano-cubosomes result in a substantial inhibition of several metabolic pathways, including AMPK/mTOR and Akt/mTOR pathways. The resultant ATP and glucose depletion leads to an increased oxidative stress and therefore apoptosis. Another mechanism for the cytotoxic effect of the nano-cubosomes is the inhibition of LDH activity which in turn results in caspase-3 activation

One of the strategies to potentiate cisplatin cytotoxicity is glucose and ATP-deprivation either through inhibition of glycolytic or mitochondrial pathways [18]. Despite the fact that cisplatin decreases glucose transporter expression and thus glucose uptake and glycolysis, our results were in accordance with Liang et al. who stated that glucose uptake is increased in cisplatin sensitive cells [19]. Another study showed that under glucose-deprivation conditions, metformin enhanced cisplatin cytotoxicity in esophageal cancer cells [20]. Metformin targets cancer cells by various mechanisms, the mitochondria being its primary target where it leads to inhibition of several complexes and hence decreased ATP production. Activated AMPK, secondary to ATP depletion, inhibits mTOR and shuts down ATP-consuming pathways to maintain energy homeostasis under cellular stress conditions. It will therefore inhibit glucose, lipid and protein synthesis needed for cell growth, whereas fatty acid oxidation [21], glucose uptake and thus glycolysis are stimulated [22, 23]. Studies revealed the amplification of chemotherapy-induced AMPK activation by metformin followed by induction of tumor cell apoptosis [24]. The current study demonstrated a significant increase in AMPK levels, secondary to decreased ATP synthesis and/or increased utilization evident by the increase in AMP/ATP ratio, in nano-cubosomes-treated groups accompanied with increased glucose uptake (Fig. 7). This effect was profound in nano-cubosomes loaded with both drugs suggesting that metformin potentiates cisplatin effect.

mTOR is one of the important kinases that is deregulated in colon cancer [25]. Its activation results in cell growth, proliferation and survival. Several chemotherapy drugs target this kinase either directly or indirectly through activation/inhibition of its upstream signaling pathways including the LKB1/AMPK/mTOR and PI3K/Akt/mTOR [26]. Experimental data showed that metformin significantly inhibited proliferation of chemo-resistant cells and its use as a neo-adjuvant chemotherapy improved patient response [24]. Apart from AMPK activation, metformin was previously reported to inhibit mTOR through Akt inactivation [27]. This dual inhibition of mTOR is advantageous since mTOR inhibitors, were reported to induce multiple resistance mechanisms, particularly feedback activation of Akt which displays abnormal signaling in colon cancer [28, 29]. Here, we found that combined cisplatin-metformin nano-cubosomes significantly inhibited p-Akt and subsequent increase in mTOR levels despite Akt upregulation, normally found in CRC. This enhanced CRC cell sensitivity to mTOR inhibition is further indicative of an important role of mTOR in cancer cell proliferation and progression.

This study also demonstrated that the drug-loaded nano-cubosomes had an initial stimulatory followed by an inhibitory effect on LDH which is a cytosolic enzyme involved in glycolysis by catalyzing the inter-conversion of pyruvate and lactate. Increased activity of LDH may be due to the observed increase in the AMPK activity that subsequently increase glucose uptake and glycolysis. Several studies demonstrated an increase in glycolysis and LDH activity after metformin or cisplatin treatment due to inhibition of the mitochondrial oxidative phosphorylation [30, 31]. Nevertheless, LDH has multiple functions in neoplastic tissues. Aberrant LDH expression is common in several tumors, promoting reliance on glycolysis, generating lactate as an end-product [32] that enhances survival, metastasis and recurrence [33, 34]. LDH overexpression is accompanied with chemo-resistance, enhancement of angiogenesis and metastasis also through the elevation of vascular endothelial growth factor [35] and metalloproteinase levels [36]. The use of cisplatin-metformin nano-cubosomes demonstrated a significant inhibition of LDH activity after 48 h of treatment coupled with an increase in NADPH oxidase activity. This signals a rise in ROS production and hence increased apoptosis (Fig. 7).

## Conclusions

In summary, cisplatin and cisplatin-metformin-loaded nano-cubosomes were successfully prepared by emulsification technique. They exhibited strong antitumor activity on human HCT-116 CRC cells in vitro compared to unformulated cisplatin. The nano-cubosomes affected several intracellular targets with significant inhibitory effect on tumorigenesis-associated metabolic pathways leading to increased apoptosis. Concisely, nano-cubosomes can be used as a potential carrier for enhancing cisplatin cytotoxicity. This cytotoxic effect can be further improved by the simultaneous presence of the indirect mTOR inhibitor, metformin, together with cisplatin in nano-cubosomal dispersions. Therefore, the prepared cisplatin and cisplatin-metformin nano-cubosomes could be potent agents in CRC treatment for further evaluation.

## Abbreviations

Akt: Protein kinase B
AMPK: Adenosine monophosphate-activated protein kinase
CRC: Colorectal cancer
ELISA: Enzyme linked immunosorbent assay
GMO: Glyceryl monooleate
LDH: Lactate dehydrogenase
LKB1: Liver kinase B1
mTOR: Mammalian target of rapamycin
NADPH: Nicotinamide adenine dinucleotide phosphate hydrogen
PI3K: Phosphoinositide-3 kinase
PVA: Polyvinyl alcohol
ROS: Reactive oxygen species
SRB: Sulphorhodamine-B
